# Editorial

**Published:** 1913-07

**Authors:** 


### EDITORIAL

The Business Office of the *Journal-Record* is 23 West Alabama Street.

The Editorial Office is 1014-15 Atlanta National Bank Building.

Address all Business Communications to *Journal-Record of Medicine,* 23 West Alabama Street.

Make remittances payable to *The Journal-Record of Medicine.*

On matters pertaining to the Editorial and Original Communications, address Edgar G. Ballenger, M. D., Atlanta, Ga.

Reprints of Original Articles will be furnished at cost price. Requests for the same should always be made in THE MANUSCRIPT.

We will present, postpaid, on request to each contributor of an original article, twenty (20) marked copies of *The Journal-Record of Medicine* containing such article.

### THE CHATTAHOOCHEE VALLEY MEDICAL AND SURGICAL ASSOCIATION.

The meeting of the Chattahoochee Valley Medical and Surgical Association was held in Montgomery, Ala. July 16th and 17th. As usual for this association, the quality of the scientific work was excellent. There were many first-class papers which provoked lively discussions. In spite of the oppressive hot weather, which made things scientific seem untimely, the attendance was good.

The spirit of cordiality and hospitality, a characteristic of these meetings, was as usual in evidence, and in behalf of the Association we beg to express the appreciation of its members for the courtesies extended by the Montgomery physicians.

Dr. Niles presided in his usual genial manner and it was with deep regret that it was learned on the second day that he had been called, by wire, back to Atlanta on account of urgent professional duties.

Dr. Poer, of West Point, Ga., was elected president for the coming year and West Point was selected as the next meeting place. Dr. McCullough was appointed on the council.

The next meeting should have an unusually large attendance as it is to be held nearly in the center of the Chattahoochee Valley territory and is surrounded by physicians who are members and who are already favorably known to the association.

